# Phenformin alone or combined with gefitinib inhibits bladder cancer via AMPK and EGFR pathways

**DOI:** 10.1186/s40880-018-0319-7

**Published:** 2018-07-27

**Authors:** Yanjun Huang, Sichun Zhou, Caimei He, Jun Deng, Ting Tao, Qiongli Su, Kwame Oteng Darko, Mei Peng, Xiaoping Yang

**Affiliations:** 1 Key Laboratory of Study and Discovery of Targeted Small Molecules of Hunan Province and Department of Pharmacy in the School of Medicine and Laboratory of Animal Nutrition and Human Health, Hunan Normal University, Changsha, 410013 Hunan P. R. China; 2 Department of Pharmacy, Xiangya Hospital, Central South University, Changsha, 410008 Hunan P. R. China

**Keywords:** Phenformin, Gefitinib, Bladder cancer, AMPK, EGFR

## Abstract

**Background:**

In previous studies, we have shown that the combination of metformin and gefitinib inhibits the growth of bladder cancer cells. Here we examined whether the metformin analogue phenformin, either used alone or in combination with gefitinib, could inhibit growth of bladder cancer cells.

**Methods:**

The growth-inhibitory effects of phenformin and gefitinib were tested in one murine and two human bladder cancer cell lines using MTT and clonogenic assays. Effects on cell migration were assessed in a wound healing assay. Synergistic action between the two drugs was assessed using CompuSyn software. The potential involvement of AMPK and EGFR pathways in the effects of phenformin and gefitinib was explored using Western blotting.

**Results:**

In MTT and clonogenic assays, phenformin was > 10-fold more potent than metformin in inhibiting bladder cancer cell growth. Phenformin also potently inhibited cell migration in wound healing assays, and promoted apoptosis. AMPK signaling was activated; EGFR signaling was inhibited. Phenformin was synergistic with gefitinib, with the combination of drugs showing much stronger anticancer activity and apoptotic activation than phenformin alone.

**Conclusions:**

Phenformin shows potential as an effective drug against bladder cancer, either alone or in combination with gefitinib.

## Introduction

The biguanide metformin is widely used as a first-line oral hypoglycemic drug against diabetes [1]. It is well tolerated and safe, with well-characterized pharmacokinetics [2, 3]. Accumulating evidence suggest that metformin could also improve the survival of patients with colorectal, breast cancer, or prostate cancer [4–6]. In fact, it may lower the risk of certain types of cancer [7]. However, obtaining approval from regulatory authorities such as the US Food and Drug Administration to use metformin as an anticancer drug has proven challenging because effective drug concentrations are much higher than anti-diabetic doses. Such high concentrations in the blood are not achievable via conventional oral administration [8, 9].

Phenformin is a derivative of metformin, with higher anticancer potency at lower doses [10–12]. Phenformin is more lipophilic than metformin, and does not require cell-surface transporters such as Oct1 to enter cells. This feature makes phenformin potentially more versatile than metformin for targeting cancer tissues, since Oct1 is not expressed in all tissues [13, 14]. Here we examined whether phenformin shows efficacy against bladder cancer [15–17]. In a previous study, we showed that the combination of metformin and the epidermal growth factor receptor (EGFR) inhibitor gefitinib produces cytotoxic effects in bladder cancer cells [9]. Gefitinib is effective as adjuvant treatment of primary bladder cancer [18]. Metformin is synergistic with gefitinib [19]. In the present study, we examined the anticancer effects of phenformin alone and in combination with gefitinib. Our goal was to determine whether phenformin could inhibit bladder cancer cell growth effectively at lower doses than metformin. Since metformin produces antitumor effects by activating adenosine monophosphate (AMP)-activated protein kinase (AMPK) and subsequent inhibition of the mTOR signaling [20–22], we also examined whether phenformin could inhibit cancer growth by activating AMPK [2, 23].

## Materials and methods

### Reagents

Phenformin (Aladdin Chemistry, Shanghai, China) was prepared in a range of concentrations in culture medium. Gefitinib (Selleck-Biotool, Shanghai, China) was prepared as a stock solution of 5 mmol/L in DMSO. Antibodies against the following target proteins were obtained from Cell Signaling (Beverly, MA, USA): total p70 S6 kinase, phospho-p70 S6 kinase (Thr389), total AMPKα, phospho-AMPKα (Thr172), total mTOR, phospho-mTOR (Ser2448), total 4E-BP1, phospho-4EBP1, total EGFR, phospho-EGFR and β-actin.

### Cell lines and culture conditions

The mouse bladder cancer cell line MB49 and the human bladder cancer cell lines T24 and UMUC3 were generously provided by Dr. P. Guo of the Institute of Urology at Xi’an Jiaotong University (Xi’an, Shaanxi, China) [9]. All cell lines were cultured in DMEM (Hyclone, Logan, UT, USA) supplemented with 10% fetal bovine serum (FBS; Hyclone) and 1% penicillin–streptomycin. Cultures were incubated at 37 °C in humidified air containing 5% CO_2_.

### Cell viability assay

Cell viability was assessed using a tetrazolium-based assay. Briefly, cells were seeded at 8 × 10^3^ per well in 96-well culture plates and incubated in medium containing 10% FBS. At 24 h later, cells were treated for 48 h with different concentrations of phenformin alone or in combination with gefitinib. The tetrazolium salt of MTT (50 μL; Sigma) was dissolved in Hank’s balanced salt solution to a concentration of 2 mg/mL, and added to each well. The plates were incubated another 5 h. The medium was aspirated from each well, DMSO (150 μL; Sigma) was added to dissolve formazan crystals, and absorbance was measured using a microplate reader (Biotek, SYNERGY HTX, VT, USA) at 490 nm (against reference absorbance at 630 nm). Dose–response curves were generated and used to calculate the half-maximal inhibitory concentration (IC_50_) using SPSS 16.0 (IBM, Chicago, IL, USA).

### Clonogenic assay

Briefly, 8 × 10^3^ cells were seeded into 24-well dishes in 0.5 mL of medium. At 24 h, cells were treated with different concentrations of phenformin alone or combined with gefitinib for a further 6–8 day period in medium containing 10% FBS. Cells were fixed with 10% formaldehyde, stained with 0.1% crystal violet. Absorbance was measured using a microplate reader (Biotek) at 550 nm wavelength. Colony formation images were captured under a microscope (DFC450C; Leica, Wetzlar, Germany).

### Cell migration

Cells (5 × 10^3^) were seeded into 6-well plates and allowed to reach confluence. The monolayer was scratched using a cocktail stick. Cells were incubated with serum-free DMEM medium for different time periods before capturing the digital images with a DFC450C microscope (Leica). Wound closure was determined by measuring the migrated distance of cells from the 0 h using Image J (US National Institutes of Health, Bethesda, MD, USA). Experiments were repeated three times.

### Apoptosis

In one set of experiments, apoptosis was assessed using fluorescence microscopy. Cells (1.2 × 10^4^) were seeded into 96-well plates. After 24 h, cells were treated with phenformin alone or with gefitinib for 24 h. Then the cells were incubated at room temperature in the dark for 15 min with 100 μL binding buffer, 1 μL FITC-conjugated Annexin V (MultiSciences Biotech, Hangzhou, China) and 1 μL of propidium iodide (MultiSciences Biotech). Cells were observed under a DFC450C fluorescence microscope (Leica).

Apoptosis was assessed using flow cytometry in a separate experiment. Briefly, cells treated with the different drug combinations were harvested with trypsinization, washed twice with phosphate-buffered saline (PBS), and resuspended in binding buffer to 1 × 10^6^ cells/mL. Then 5 μL of Annexin V-FITC and 10 μL of propidium iodide were added to 100 μL of cell suspension, incubated for 30 min at room temperature in the dark, and then mixed with 400 μL of binding buffer. Within 30 min, labeled cells were counted by flow cytometry on a FACS Calibur flow cytometer [excitation wavelength, 488 nm; emission wavelengths, 530 nm (FL-1 channel, FITC) and 670 nm (FL-3 c3 channel, propidium iodide)]. Data were analyzed using Cell Quest software (Becton–Dickinson). Non-apoptotic cells were defined as those negative for Annexin-V and propidium iodide; necrotic/late apoptotic cells as those positive for both labels; and early apoptotic cells as those positive for Annexin V but negative for propidium iodide.

### Western blotting

Proteins were fractionated by SDS-PAGE, transferred to membranes, and then incubated overnight at 4 °C with different primary antibodies described in Reagents section above (Cell Signaling, Beverly, MA, USA) in buffer containing bovine serum albumin (BSA). Membranes were washed with TBS containing 0.05% Tween-20, blotted with secondary antibody for 1 h at room temperature, then washed again three times. Pierce Super Signal chemiluminescent substrate (Rockford, IL, USA) was added, and the blot was imaged immediately on a Chemi Doc system (Bio-Rad, Hercules, CA, USA) and a Perfection V500 camera (Epson). Band intensities were quantified using Image J.

### Statistical analyses

All data are presented as mean ± SD. Statistical analysis was performed using SPSS 16.0. Differences between groups were assessed for significance using Student’s *t* test for experiments involving only two groups and using ANOVA and the least significant difference (LSD) test for experiments involving more than two groups. Graphs were generated using GraphPad Prism 6.0. Two levels of statistical significance were considered: *P < 0.05 and ^#^*P *< 0.01.

## Results

### Phenformin inhibits bladder cancer cell proliferation

**Fig. 1:**
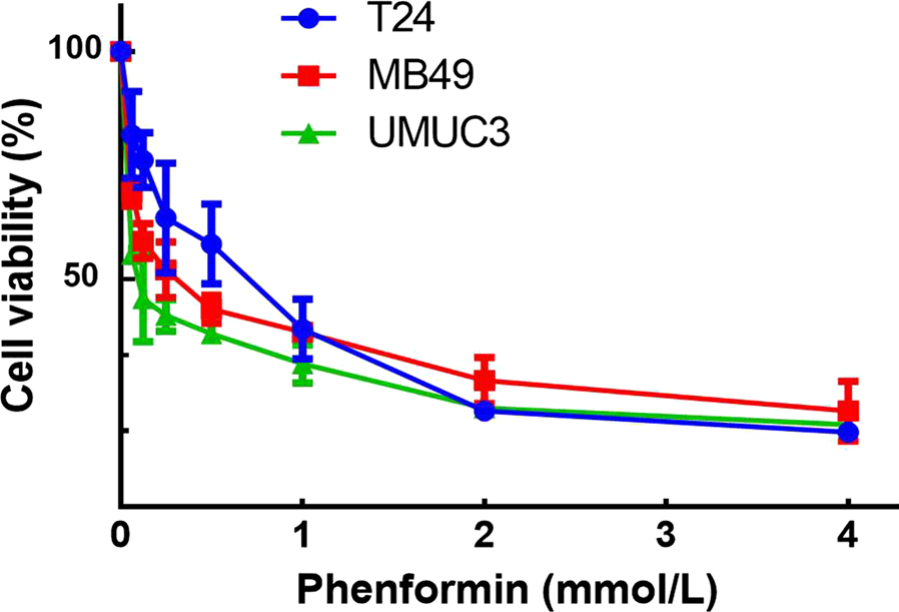
Effect of phenformin on bladder cancer cell proliferation. Viability of T24, UMUC3 and MB49 cells was assessed after 48-h exposure to phenformin at concentrations ranging from 0 to 8 mmol/L using a tetrazolium-based assay. Results are the mean ± SD of three independent experiments

Phenformin inhibited cell proliferation within a concentration range of 0.02–8 mmol/L in a concentration-dependent manner (Fig. 1). The most sensitive cell line was UMUC3 (IC_50_, 0.25 mmol/L); the most resistant cell line was T24 ((IC_50_, 0.87 mmol/L; Table 1).

**Table 1:** IC_50_ values for phenformin against bladder cancer cell lines

	Bladder cancer cell line		
	MB49	T24	UMUC3
IC_50_ (mmol/L)	0.57	0.97	0.25

### Phenformin synergistically potentiates the anti-proliferative effects of gefitinib

**Fig. 2:**
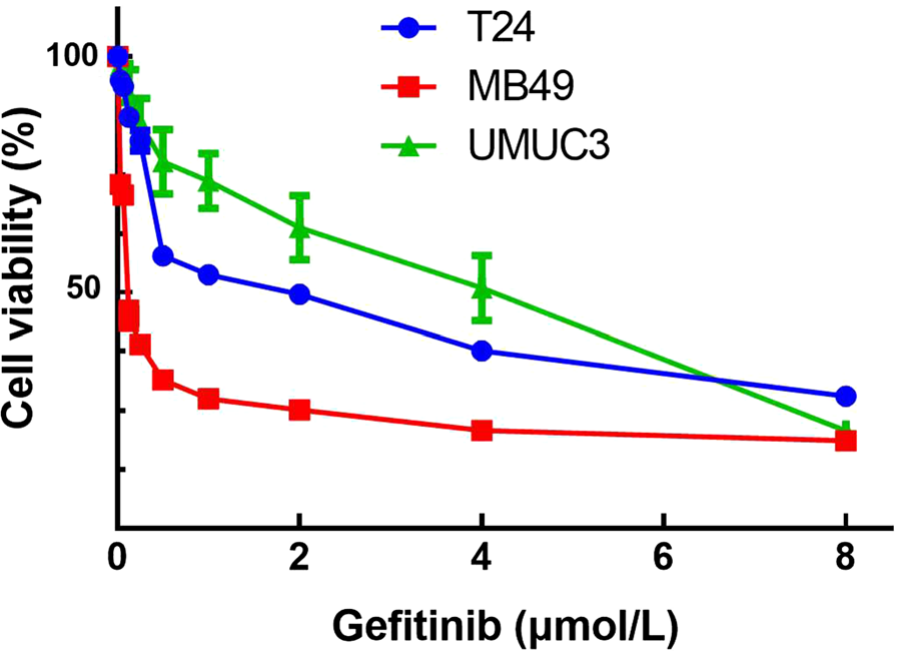
Effect of gefitinib on bladder cancer cell proliferation. Viability of T24, UMUC3 and MB49 cells was assessed after 48-h exposure to gefitinib at concentrations ranging from 0 to 80 µmol/L using a tetrazolium-based assay. Results are the mean ± SD of three independent experiments

**Fig. 3:**
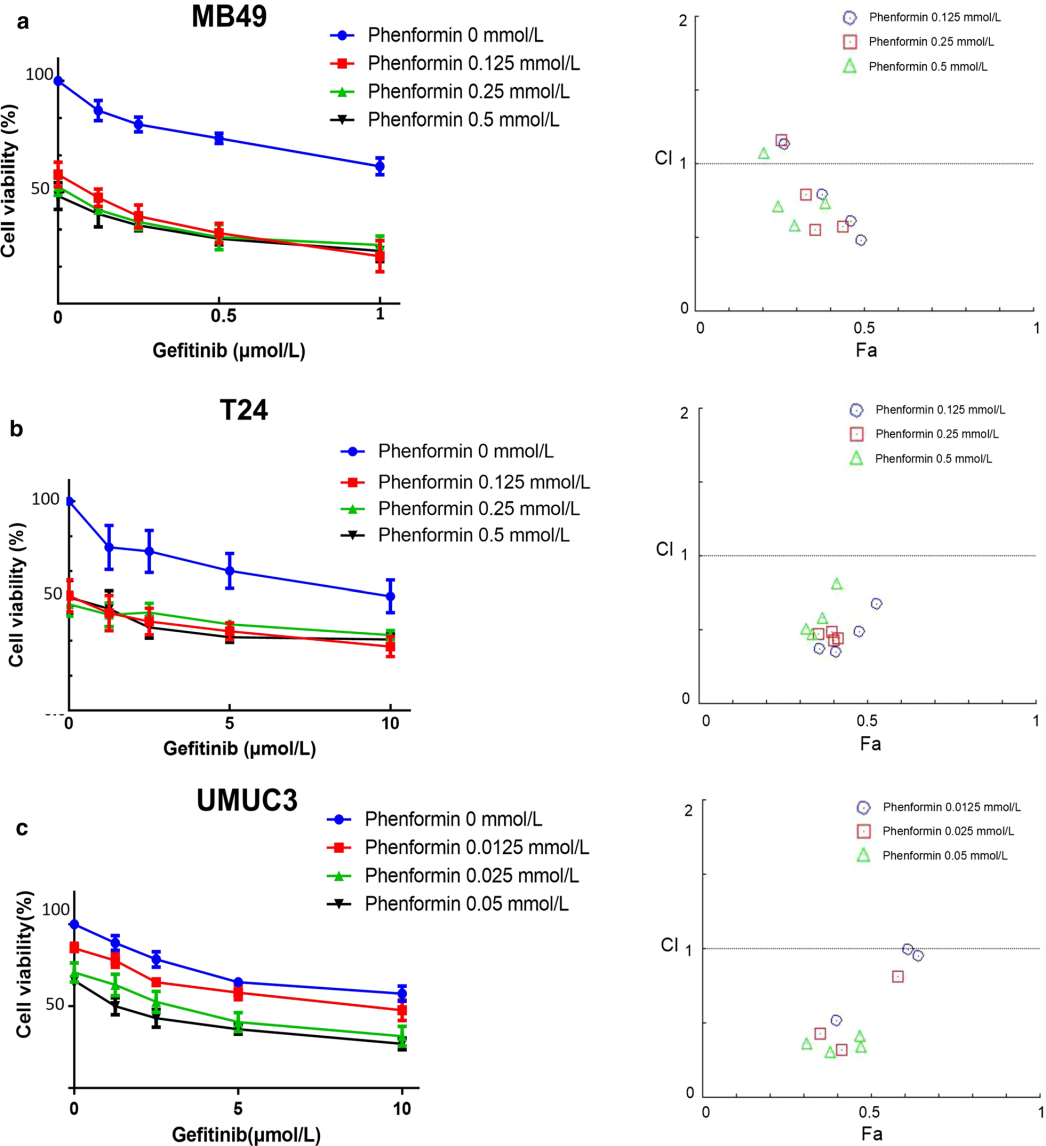
Effect of gefitinib combined with phenformin on bladder cancer cell proliferation. **a** Gefitinib combined with phenformin inhibited MB49 proliferation synergistically. Above: Cell viability was assessed after 48-h treatment with gefitinib (0, 0.125, 0.25, 0.5, or 1 µmol/L) alone or combined with phenformin (0.125, 0.25, or 0.5 mmol/L). Below: The combination index (CI) assessing synergy between the two drugs was calculated. **b** Gefitinib combined with phenformin inhibited T24 proliferation synergistically. Above: Cell viability was assessed after 48-h treatment with gefitinib (0, 1.25, 2.5, 5, or 10 μM) alone or combined with phenformin (0.125, 0.25, or 0.5 mmol/L). Below: The combination index (CI) assessing synergy between the two drugs was calculated. CI = 1 denotes additivity; CI > 1, antagonism; CI < 1, synergism. CI values in nearly all combinations were less than 0.5, indicating moderately strong synergism. **c** Gefitinib combined with phenformin inhibited UMUC3 proliferation synergistically. Above: Cell viability was assessed after 48-h treatment with gefitinib (0, 1.25, 2.5, 5, or 10 µmol/L) alone or combined with phenformin (0.0125, 0.025, or 0.05 mmol/L). Below: The combination index (CI) assessing synergy between the two drugs was calculated. Results are the median of five independent experiments

Consistent with our previous study [9], gefitinib did not affect the growth of the cell lines (Fig. 2, Table 2), but interacted synergistically with phenformin to inhibit cancer cell growth (Fig. 3).

**Table 2:** IC_50_ values for gefitinib against bladder cancer cell lines

	Bladder cancer cell line		
	MB49	T24	UMUC3
IC_50_ (µmol/L)	1.6	30.1	17

### Phenformin alone or combined with gefitinib suppresses colony formation

**Fig. 4:**
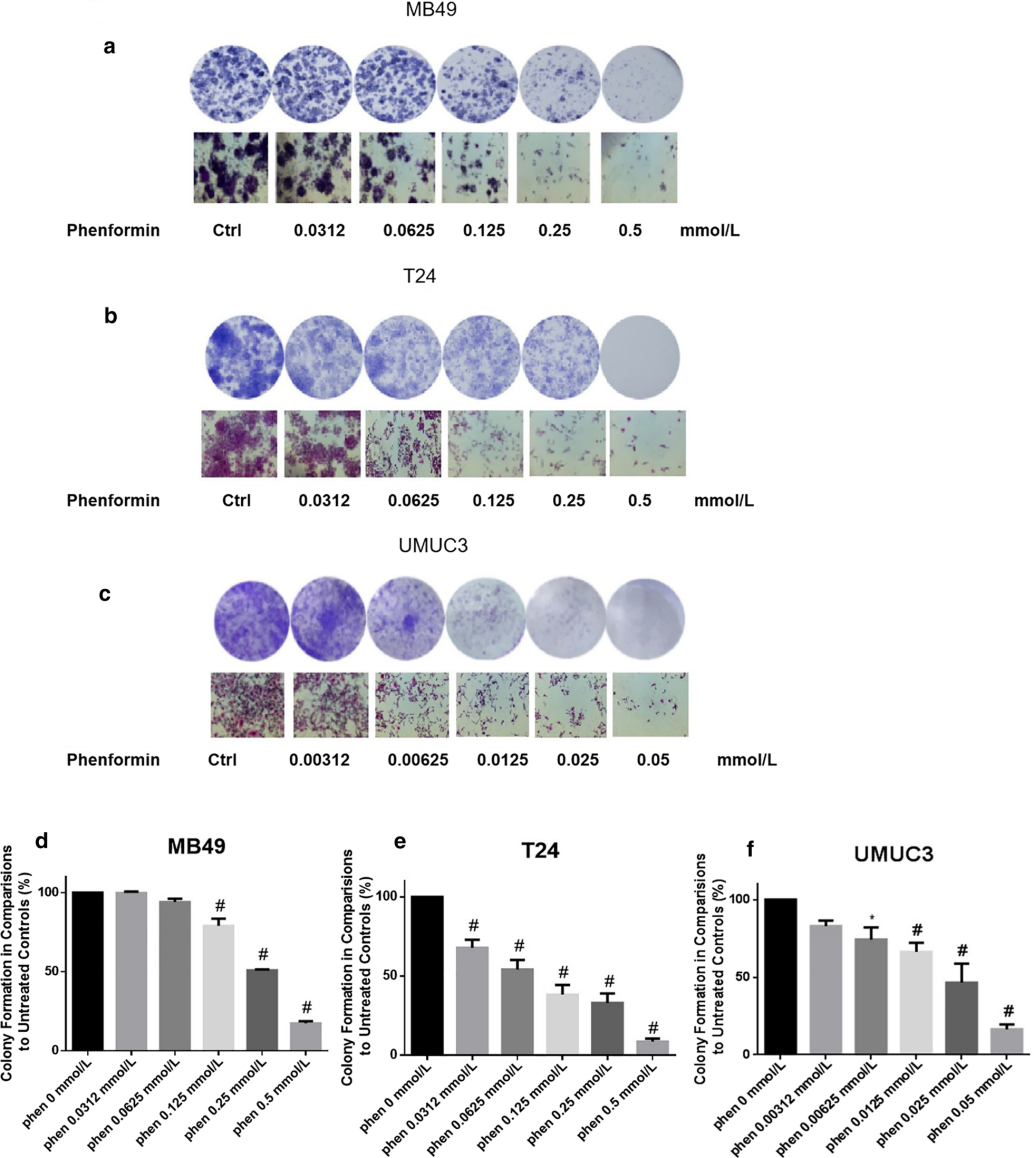
Evaluation of colony suppression by phenformin on three bladder cancer cell lines. **a**–**c** Cells were treated for 7 days with phenformin alone, and then stained with crystal violet to allow colony counting. MB49 and T24 cells were treated with 0–0.5 mmol/L phenformin; UMUC3 cells, with 0–0.05 mmol/L. Wells were photographed using an inverted microscope (magnification, ×10). Control wells contained no drug. **d**–**f** Quantification of the experiments conducted in panels (**a**–**c**). Wells were scanned at a wavelength of 550 nm. Results are the mean ± SD of five independent experiments. *P < 0.05, ^#^*P* < 0.01 vs. control (two-tailed *t* test)

**Fig. 5:**
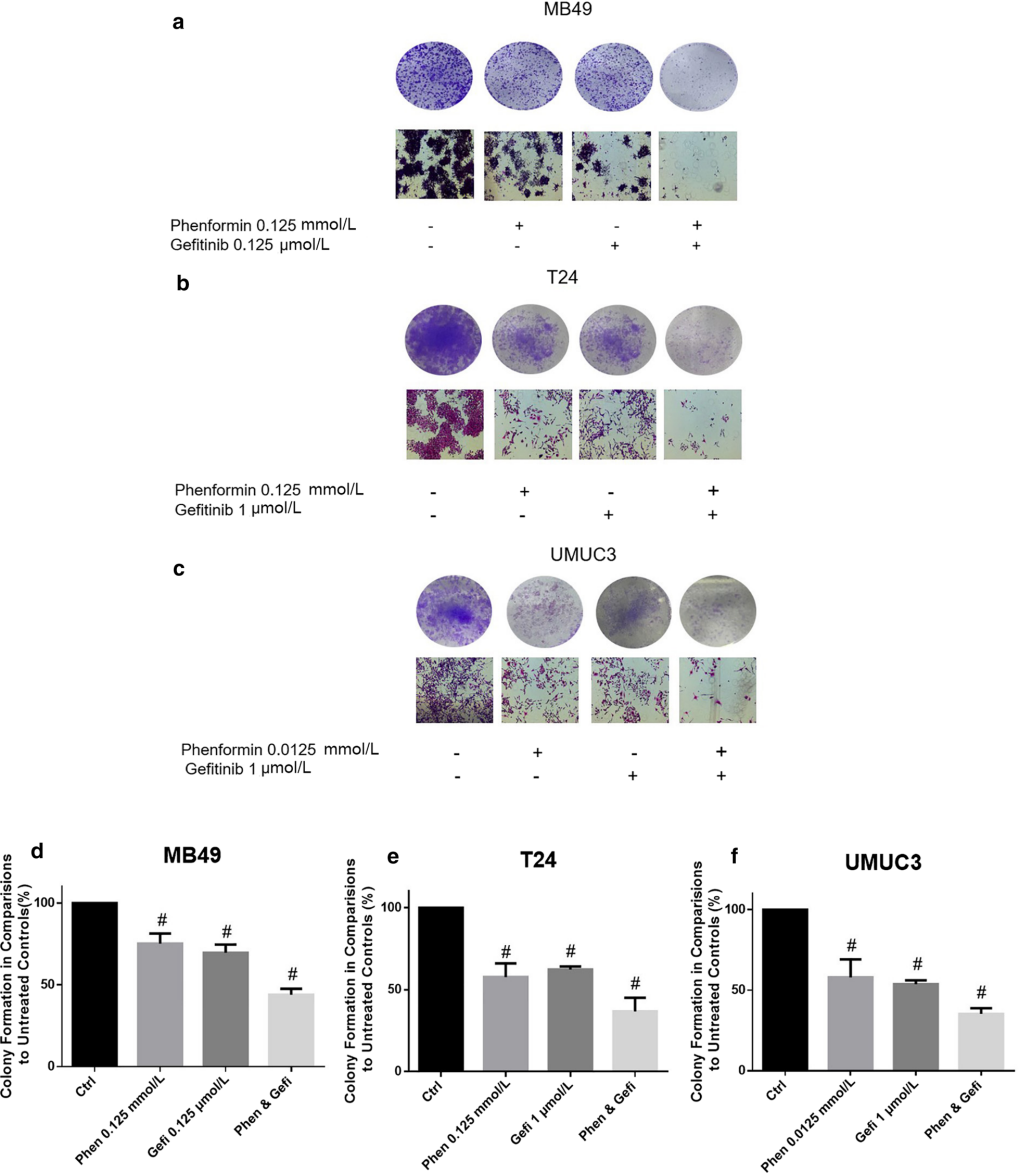
Evaluation of colony suppression by phenformin combined with gefitinib. **a**–**c** Cells were treated for 7 days with phenformin alone, gefitinib alone or both, and then stained with crystal violet to allow colony counting. MB49 cells were treated with 0.125 mmol/L phenformin, 0.125 µmol/L gefitinib, or both; T24 cells, with 0.125 mmol/L phenformin, 1 µmol/L gefitinib, or both; UMUC3 cells, with 0.0125 mmol/L phenformin, 1 µmol/L gefitinib, or both. Wells were photographed using an inverted microscope (magnification, ×10). Control wells contained no drug. **d**–**f** Quantification of the experiments conducted in panels (**a**–**c**). Wells were scanned at a wavelength of 550 nm. Results are the mean ± SD of five independent experiments. *P < 0.05, ^#^*P* < 0.01 vs. control (two-tailed *t* test)

Phenformin alone inhibited colony formation in all three cell lines at concentrations of 0–0.5 mmol/L (Fig. 4). At a concentration as low as 0.125 mmol/L, phenformin significantly reduced the numbers of T24 and MB49 colonies. The most sensitive cell line was UMUC3. Combining phenformin with gefitinib led to an even greater inhibition (Fig. 5).

### Phenformin alone or combined with gefitinib inhibits cell migration

**Fig. 6:**
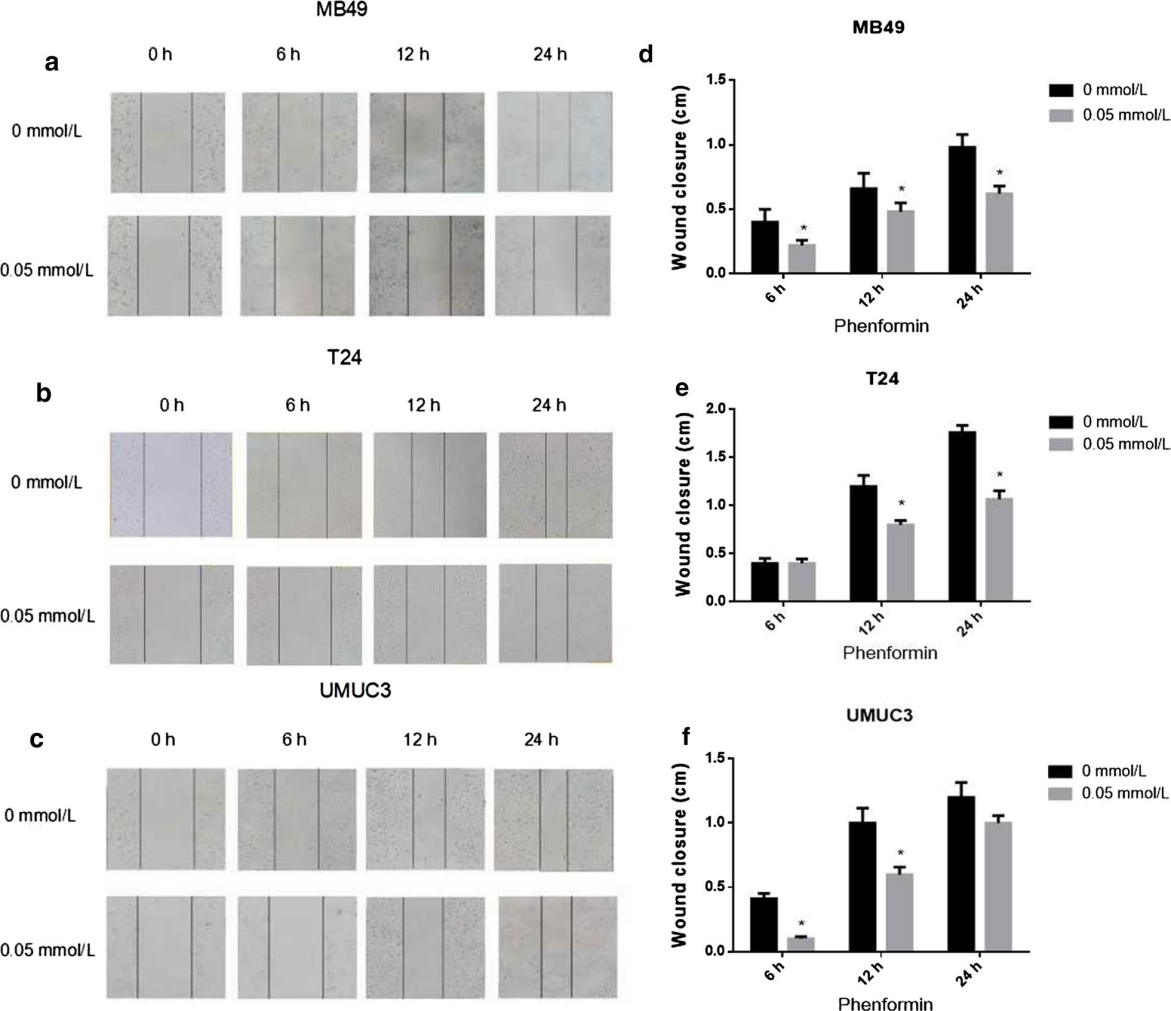
Phenformin impaired cellular migration in wound healing assays. **a**–**c** Photographs showing gaps in the scratched regions of MB49, T24, and UMUC3 monolayers subsequently treated with the indicated drug. Control wells received no drug. **d**–**f** Wound closure distances measured from the experiments shown in panels (**a**–**c**). Results are mean ± SD of three independent experiments. *P < 0.05, ^#^*P* < 0.01 vs. control

**Fig. 7:**
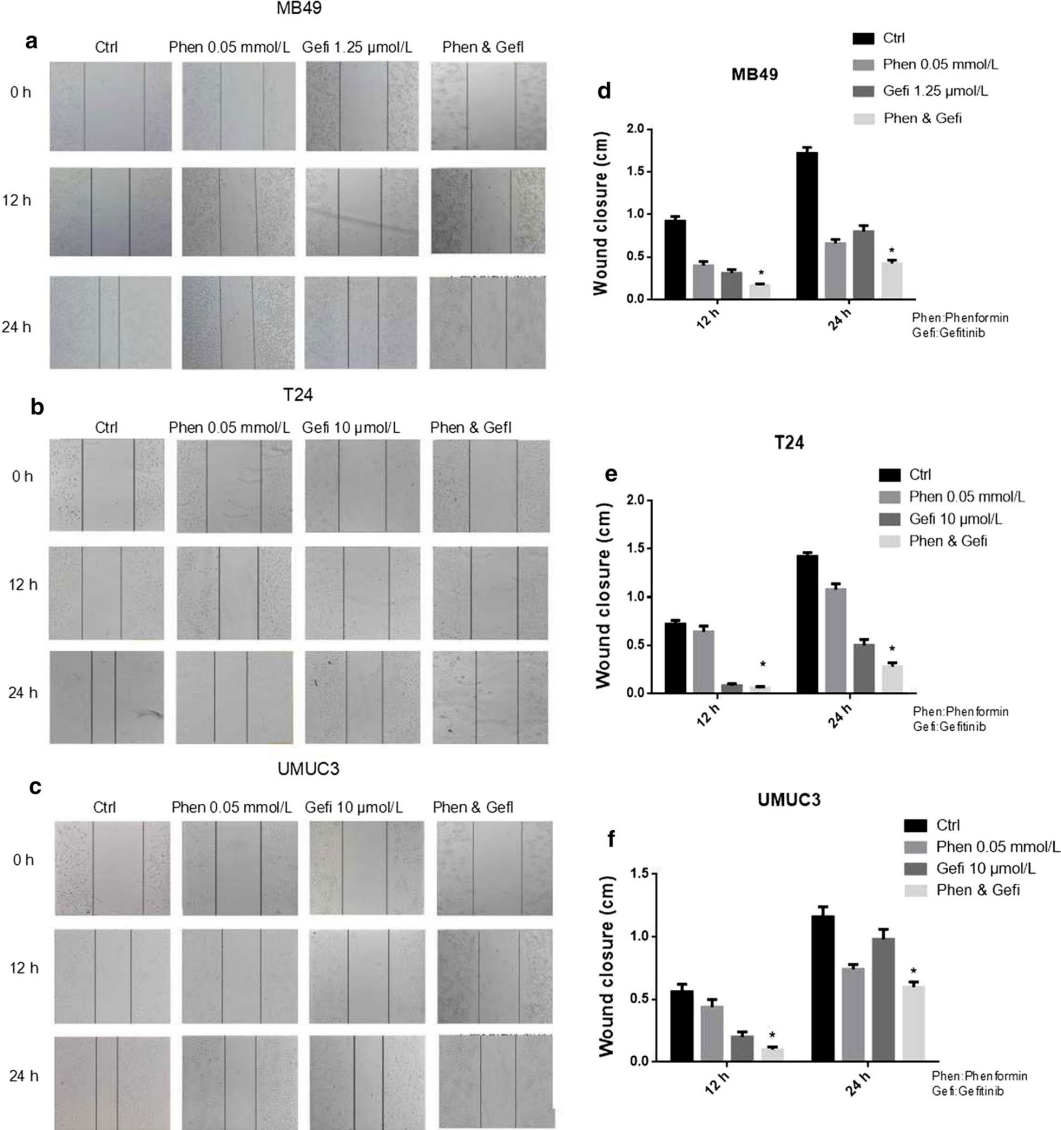
Phenformin treatment combined with gefitinib impaired cellular migration in wound healing assays. **a**–**c** Photographs showing gaps in the scratched regions of MB49, T24, and UMUC3 monolayers subsequently treated with the indicated drug alone or combination. Control wells received no drug. **d**–**f** Wound closure distances measured from the experiments shown in panels (**a**–**c**). Results are mean ± SD of three independent experiments. *P < 0.05, ^#^*P* < 0.01 vs. control

In the scratch assay, phenformin significantly increased the cell-free area at 24 h (*P* < 0.05 vs. control, Fig. 6). The assay was repeated by treating MB49 cells with 0.05 mmol/L phenformin alone, 1.25 μmol/L gefitinib alone or their combination. In addition, the assay was repeated by treating T24 and UMUC3 cells with 0.05 mmol/L phenformin alone, 10 μmol/L gefitinib alone or their combination. The combination of drugs inhibited migration to a greater extent than either drug alone (Fig. 7).

### Phenformin alone or combined with gefitinib activates AMPK signaling pathways

**Fig. 8:**
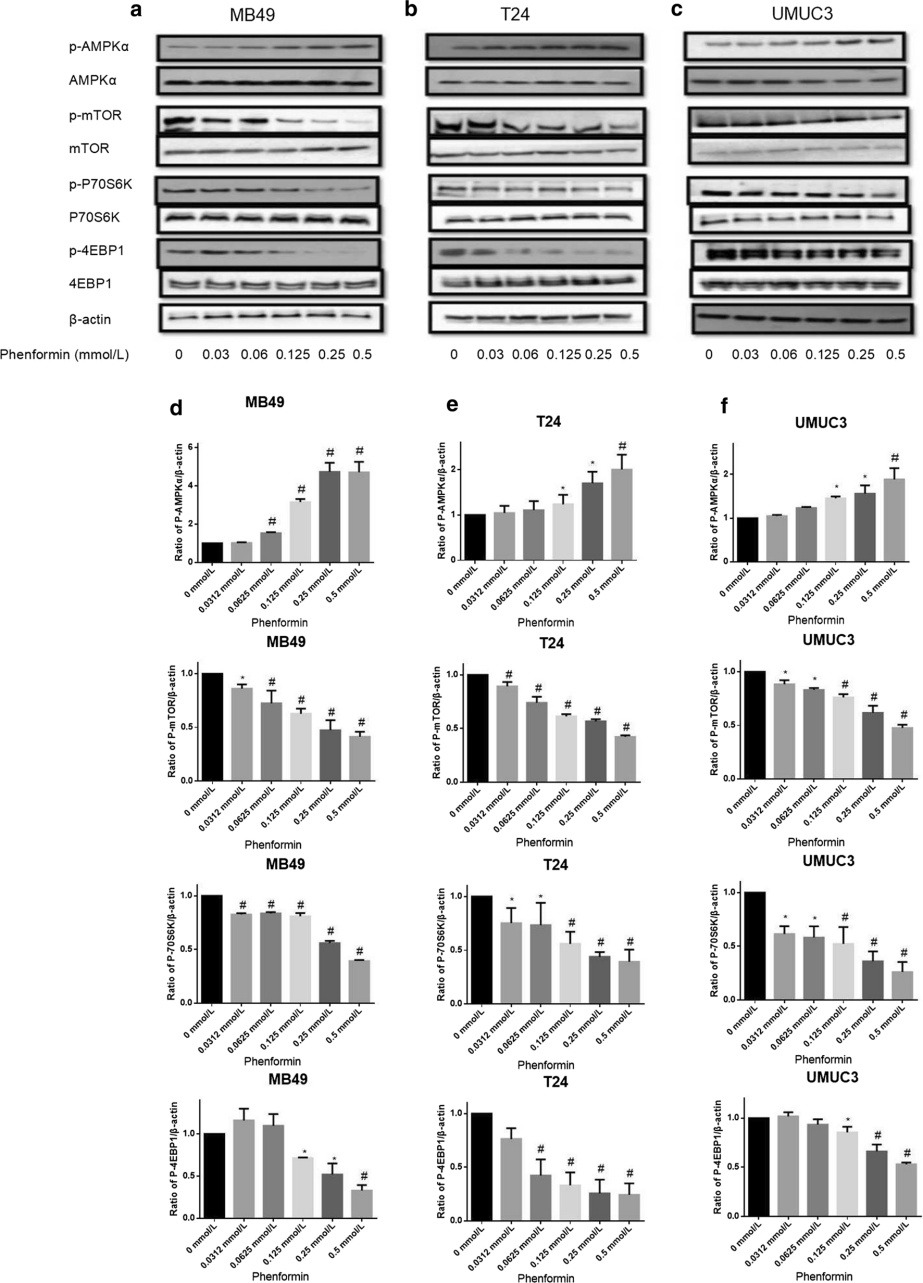
Effects of phenformin on AMPK intracellular signaling pathways. Western blot analysis was used to examine the levels of total (t) and phosphorylated (p) forms of various signaling proteins in MB49, T24, and UMUC3 cells treated with phenformin at various concentrations. Control cells received no drugs. **a**–**c** Western blots of p-AMPK, p-mTOR, p-p70S6K, p-4EBP1, t-AMPK, t-mTOR, t-p70S6K and t-4EBP1. β-actin was included as a loading control. **d**–**f** Relative levels of various proteins. Results are mean ± SD. *P < 0.05, ^#^*P* < 0.01 vs. control

All three cell lines were treated with phenformin at doses of 0–0.5 mmol/L for 36 h, and cell lysates were analyzed by Western blotting for levels of p-AMPK (T172), p-mTOR (S2448) and the mTOR downstream effectors p70S6K and p4EBP1 (Fig. 8). Phenformin activated AMPK and led to lower levels of phosphorylated 4EBP1 and p70S6K. In our previous work, we observed similar effects with metformin but only at much higher concentrations [9].

**Fig. 9:**
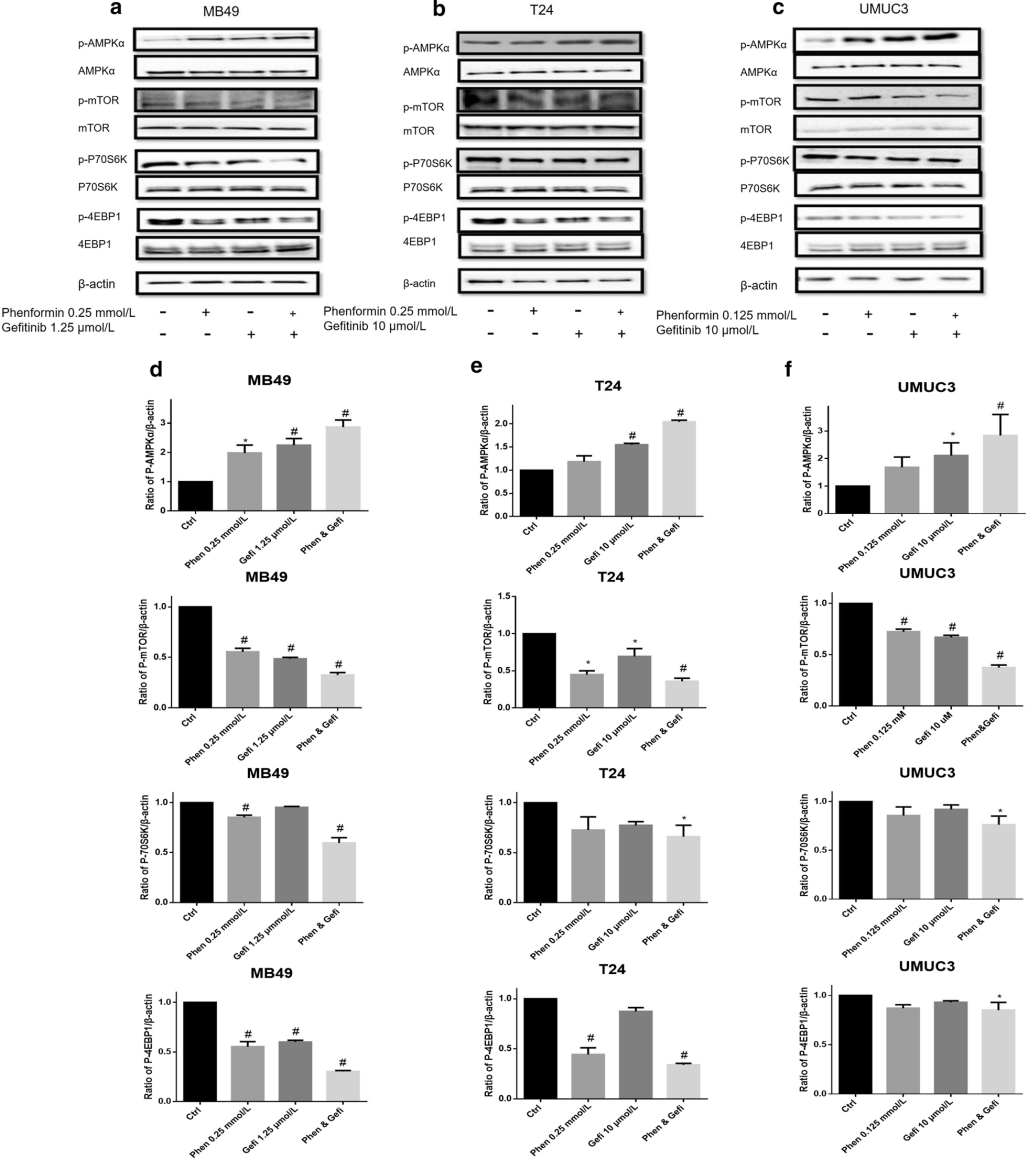
Effects of the combination of gefitinib with phenformin on AMPK signaling pathways. Western blot analysis was used to examine total (t) and phosphorylated (p) forms of various signaling proteins in MB49, T24, and UMUC3 cells treated with phenformin, gefitinib or their combination. Control cells received no drugs. **a**–**c** Western blots of p-AMPK, p-mTOR, p-p70S6K, p-4EBP1, t-AMPK, t-mTOR, t-p70S6K and t-4EBP1. β-actin was included as a loading control. **d**–**f** Relative levels of various proteins. Results are mean ± SD. *P < 0.05, ^#^*P* < 0.01 vs. control

AMPK signaling pathway activation was much higher upon treatment with phenformin in combination with gefitinib than with either drug alone (Fig. 9). In our previous work [9], we achieved strong synergy with the combination of metformin and gefitinib, but at a metformin concentration eightfold higher than the phenformin concentration in the present work. The synergy between phenformin and gefitinib was greatest for the UMUC3 cell line.

### Phenformin alone or combined with gefitinib inhibits the EGFR signaling pathways

**Fig. 10:**
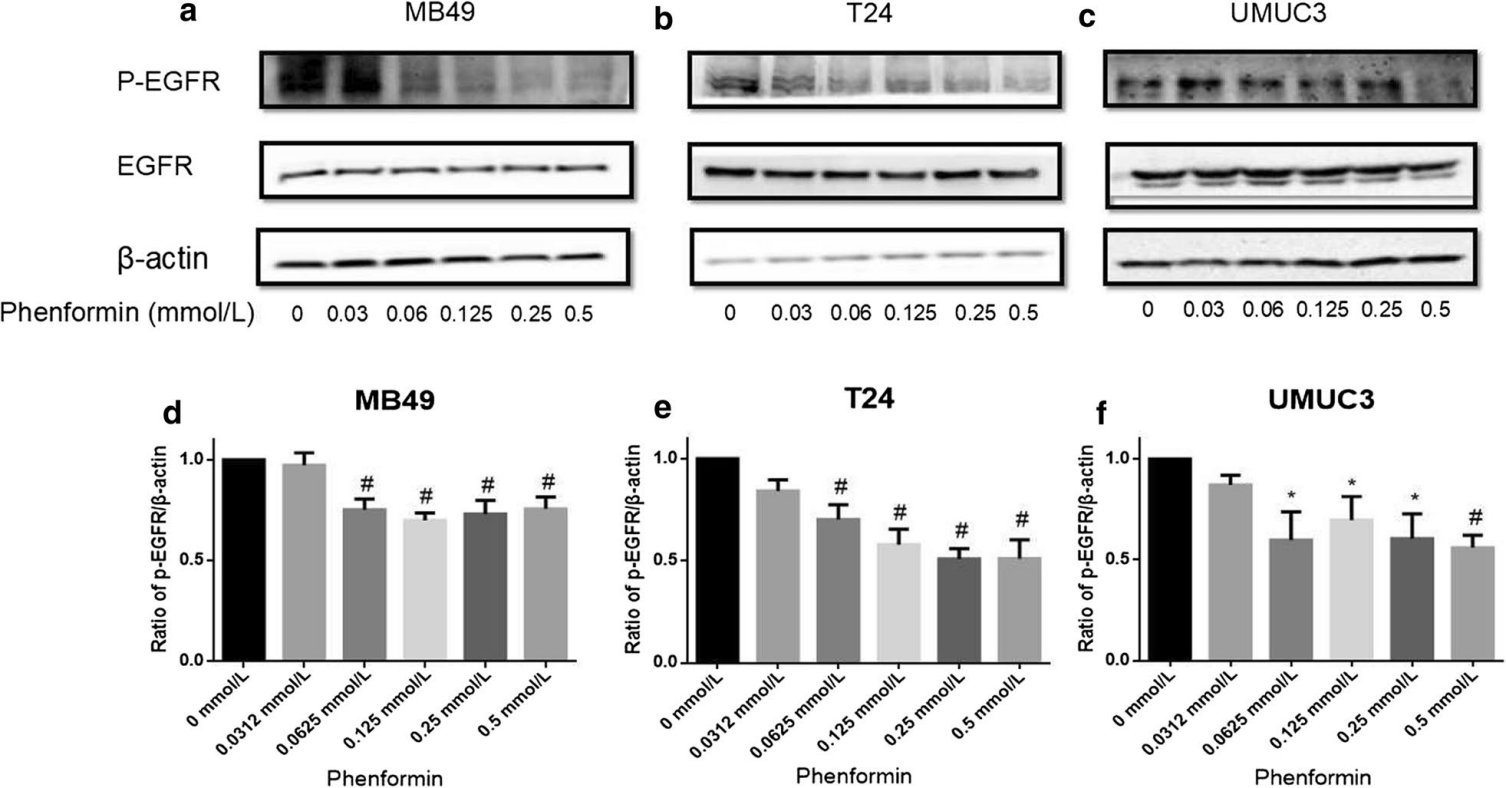
Effects of phenformin on EGFR phosphorylation. Western blot analysis was used to examine total (t) and phosphorylated (p) forms of EGFR in MB49, T24, and UMUC3 cells treated with phenformin at various concentrations s. **a**–**c** p-EGFR and t-EGFR. β-actin was included as a loading control. **d**–**f** Relative levels of phosphorylated EGFR. Results are mean ± SD. *P < 0.05, ^#^*P* < 0.01 vs. control

**Fig. 11:**
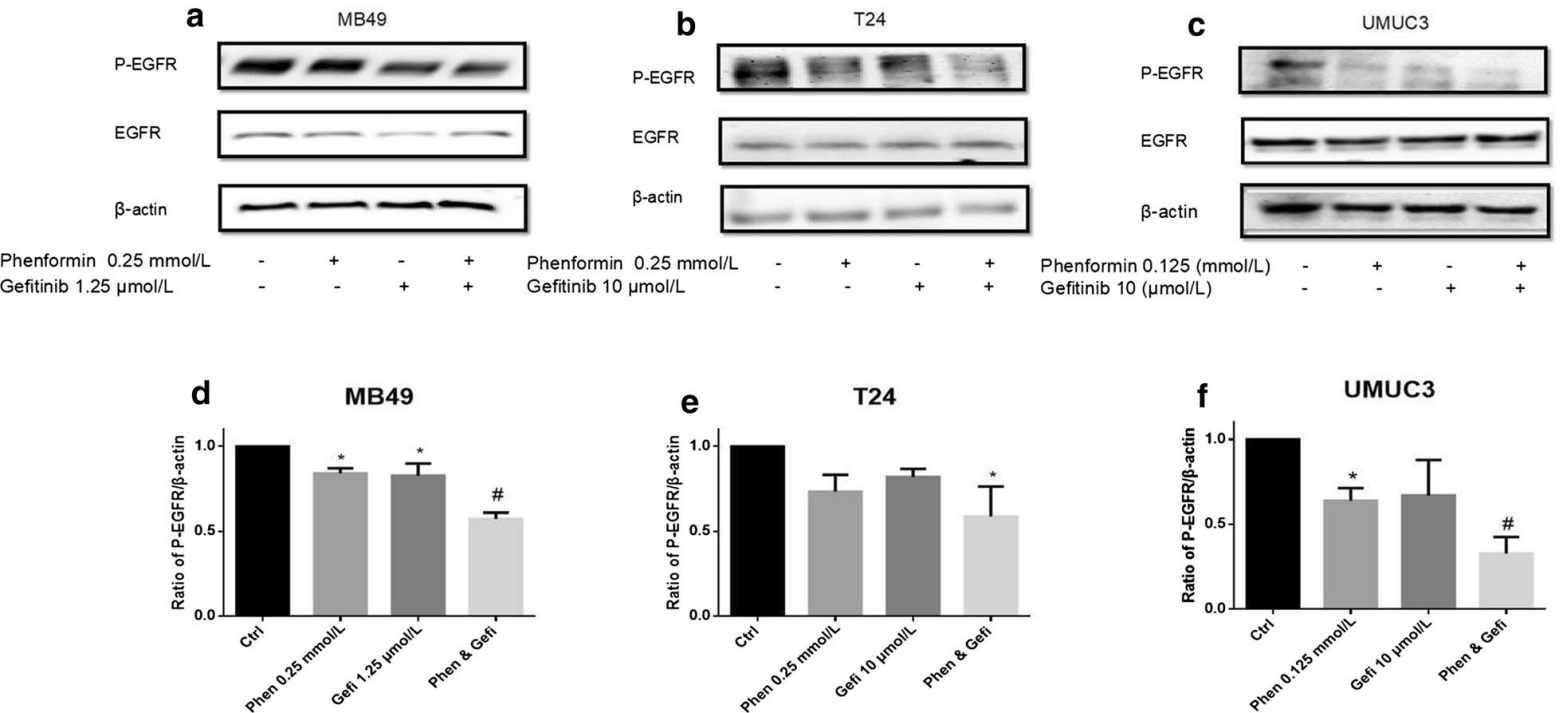
Effects of the combination of gefitinib with phenformin on EGFR phosphorylation. Western blot analysis was used to examine total (t) and phosphorylated (p) forms of EGFR in MB49, T24, and UMUC3 cells treated with phenformin, gefitinib or their combination. **a**–**c** Western blotting of p-EGFR and t-EGFR. β-actin was included as a loading control. **d**–**f** Relative levels of phosphorylated EGFR. Results are mean ± SD. *P < 0.05, ^#^*P* < 0.01 vs. control

In our previous work [19], we showed that metformin and gefitinib cooperatively inhibited bladder cancer growth by inhibiting EGFR signaling. Here we examined whether the same was true for the combination of phenformin and gefitinib (Figs. 10 and 11). We found that phenformin alone or together with gefitinib inhibited EGFR signaling at concentrations eightfold lower than used in our previous work.

### Phenformin alone or combined with gefitinib promotes apoptosis

**Fig. 12:**
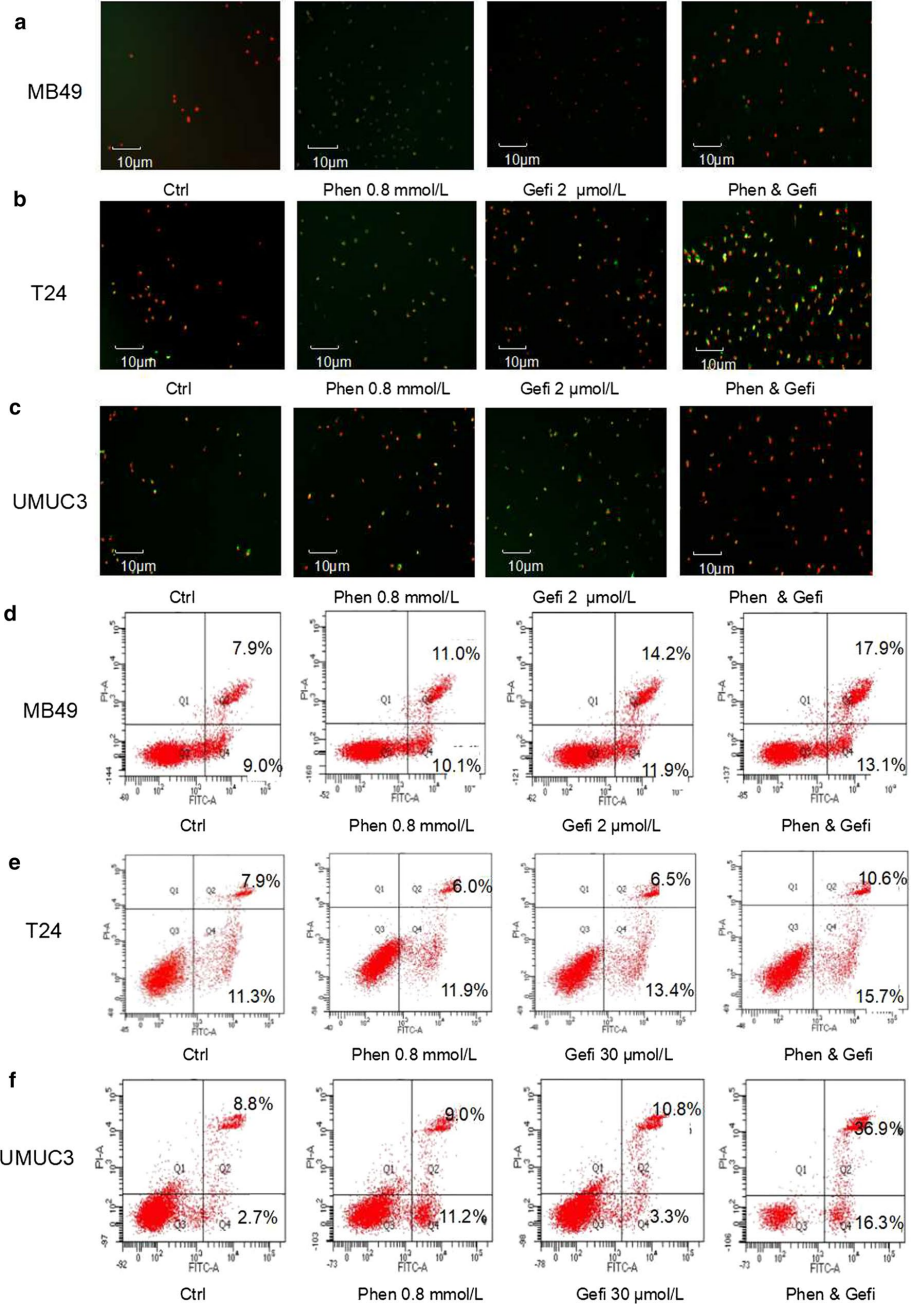
Effect of gefitinib combined with phenformin on apoptosis. **a**–**c** Fluorescence micrographs to assess FITC and propidium iodide staining of MB49, T24, and UMUC3 cell lines treated with the indicated drugs. Control cells received no drugs. **d**–**f** Representative flow cytometry scatter plots showing propidium iodide (*y* axis) and Annexin V-FITC (*x* axis) staining. Quantitation of flow cytometry experiments. Results are the mean ± SD of three independent experiments. *P < 0.05, ^#^*P* < 0.01 vs. control

Either phenformin or gefitinib alone substantially increased the proportion of apoptotic cells in all three cell lines (Fig. 12). The combination of the two drugs led to a much higher proportion of apoptotic cells than either drug alone.

## Discussion

The results from the current study showed that phenformin, either alone or in combination with gefitinib, could produce antitumor effects in bladder cancer cells. Phenformin may be better suited for this purpose than its parent compound metformin. Metformin has been shown to inhibit bladder cancer cell proliferation in vitro and in vivo [9], but only at concentrations that are difficult or impossible to achieve in human subjects. In addition, a trial in patients with type 2 diabetes failed to find an association between the use of metformin and decreased incidence of bladder cancer [24].

Our results in the present study suggest that phenformin can substantially inhibit bladder cancer cell proliferation, colony formation and migration at much lower concentrations than metformin. For example, phenformin inhibited colony formation in the most sensitive UMUC3 cell line by > 100-fold greater than metformin at tenfold lower concentrations. These cellular effects at much lower phenformin concentrations were associated with the activation of AMPK signaling and inhibition of EGFR signaling. Our findings extended the literature of phenformin antitumor activities from breast cancer cells and other cell types to bladder cancer [25].

Based on previous work [19, 26], we speculate that the much higher therapeutic efficiency of phenformin over metformin can be attributed to higher lipophilicity of phenformin and the fact that phenformin does not require organic cation transporters to enter cells [27]. Such transporters are not expressed in all tissues. As a result, phenformin can readily enter a broader range of cell types.

Metformin and phenformin increase AMPK activity without increasing the AMP/ATP ratio [28], which is important for the anti-cancer mechanism of both biguanides [9]. Tumorigenesis is a multistep process and tumor cells often undergo metabolic re-programming to support the rapid growth [29]. Targeting metabolic re-programming using biguanides represents a promising therapeutic strategy in cancer. In the present study, we showed that phenformin activates AMPK phosphorylation in bladder cancer cells. We also demonstrated that phenformin inactivates two proteins that act downstream of AMPK, namely 4EBP1 and p70S6K. These results suggest that phenformin may induce apoptosis in bladder cancer cells via the AMPK/mTOR/p70S6K axis.

Targeting multiple sites, such as with phenformin and gefitinib is generally superior to single-target anticancer therapy. The synergistic action observed in the current study probably reflects mechanistic “crossover”: we showed here that phenformin inhibits EGFR signaling in a dose-dependent manner, and we previously showed that gefitinib can activate AMPK signaling [19]. As a result of this synergy, the combination of the two drugs inhibited bladder cancer cell proliferation and colony formation while stimulating apoptosis to a much greater extent than either drug alone.

Our in vitro findings here should be verified and extended in preclinical animal studies. In addition to efficacy, whether the combination therapy is associated with toxic effects in vivo should be examined. Despite these limitations, the present work provides evidence that phenformin can effectively inhibit bladder cancer growth by activating AMPK signaling and inhibiting EGFR signaling. The results also encourage future studies to explore phenformin on its own and combined with gefitinib as multi-targeted anticancer therapy.
